# Massachusetts Dental Society

**Published:** 1899-11

**Authors:** 


### MASSACHUSETTS DENTAL SOCIETY.

The Thirty-fifth Annual Meeting of the Massachusetts Dental Society was held at Boston, June 7 and 8, 1899, the President, Harry S. Draper, D.D.S., of Boston, in the chair. The session opened on Wednesday morning, June 7, at ten o'clock with Councillors' Meeting for the transaction of routine business and the election of officers for the ensuing year.

The President called the meeting to order, and after listening to Secretary Kinsman's report of the Thirty-fourth Annual Meeting the President delivered his annual address.

MEMBERS OF THE MASSACHUSETTS DENTAL SOCIETY,—At this the close of the fourth year of the subdivision of the Massachusetts Dental Society into districts, it would seem timely, in making a report of the condition of this and the district societies, as required by the by-laws, to call attention to the conclusions that may be deduced from our experience under the new system.

It was expected by those who were instrumental in bringing about this change that a fresh stimulus would be given by holding more frequent meetings in a more social way, but after a slight influx of members at the start, there seems to be once more a general apathy exhibited, and affairs, with one or two notable exceptions, have come to a stand-still. Several of the district societies do not meet at all, and have not even elected their boards of councillors; the largest one has met only twice since the last annual meeting. What is the cause of this indifference, and where is the remedy?

The board of councillors, consisting of thirty-five members according to the by-laws, was at an early date found to be too unwieldy to perform its duties of providing places for meetings, essayists, subjects for discussion, etc., and it was reduced in size by selecting one councillor from each district to constitute an Executive Committee, thus returning to the manner in vogue before the Society was subdivided. Even then it was impossible to secure the attendance of the members of this committee from the more distant parts of the State, and matters of importance were left to one or two members to arrange. This condition is worse than when the Society was undivided, as at that time the Executive Committee could be selected from members who were near at hand and would attend committee meetings.

It was thought that it would be an easy way to secure material for the annual meetings by selecting the more meritorious essays read at the metings of the district societies, but when the district societies do not meet at all, or only occasionally, this cannot be considered an available supply.

The reports of the condition of the various districts, as received from the secretary, are as follows:

NORTH METROPOLITAN.

Two meetings have been held at Lynn. At the last one Dr. Chiconi read a paper on Tuberculosis. The officers have been elected, also one new member.

SOUTH METROPOLITAN.

A meeting was held at the American House, October 24, 1898. Dr. H. A. Lothrop, of Boston, read a paper, illustrated with dry and moist specimens, on "The Etiology and Treatment of Empyema of the Antrum of Highmore." The December and February meetings were not held, as we were disappointed by the essayists at the last moment. The annual meeting was held at the American House, May 1, 1899. John H. Coffin, M.D., read a paper on "Syphilis of the Mouth." Nine new members were admitted and officers were elected. Three members were dropped for non-payment of dues. The society now has seventy-one active members, besides eleven new members who have not signed the constitution and by-laws.

NORTHEASTERN.

No meetings, and no officers elected for several years past. Have lost the list and forgotten who the officers last elected were.

SOUTHEASTERN.

No meetings during the past year. No officers elected. There are nineteen members.

CENTRAL.

One meeting during the year, at which one new member was admitted and officers were elected. Fourteen members.

VALLEY.

Forty-two members. Officers elected and meetings held the third Monday evening of every month except July and August. Papers by Dr. Clark, of Northampton, C. S. Hurlburt, Jr., and W. H. Cummings, of Springfield, Dr. A. C. Hart, San Francisco, and discussion on incidents of practice, etc.

This district is the notable exception referred to previously.

WESTERN.

No annual meeting and consequently no election of officers. Tried to have Executive Committee prepare a programme for an annual meeting, without success. Two meetings held since last report with an average attendance of one and a half. Twenty-one members. Five papers, as follows: Toothpick Discussion, Dr. Wilder; Orthodontia Radical, Dr. G. C. Hubbell; *résumé* of paper by Dr. Leon Williams, "The Empirical Method," Dr. S. S. Stowell, Dr. Schillinger, Dr. E. S. Davenport.

I have also a letter from one of the district treasurers, who says he has not collected any dues because he considered the district society a farce, and did not feel right about asking people to pay money into an apparently defunct organization.

These reports show a lamentable lack of interest in the welfare of the State Society. In at least one district a new independent local society has been started during the past year, dentists seeming to prefer this to the district society. In Boston there are so many local societies that there is not as much enthusiasm shown in regard to the State and district societies as there might be otherwise. This indifference has of course militated against the success of the Massachusetts Society, which was expected, by reason of the reorganization, to become a powerful factor throughout the State for the advancement of the profession, the enforcement of dental laws, etc. It has been suggested that if it were fully shown that the Society did have a decided influence in checking illegal practice, more of the reputable practitioners would become members. As the dental law now stands, there is no provision for prosecuting its violators, and, although their attention may be called to cases of illegal practice, it is too much to expect that those who were merely appointed to examine and license candidates should also spend their time, without remuneration, in convicting violators of the laws. There being no one to attend to the looking up of evidence and prosecuting cases, it would seem desirable that the State Society should look after this matter; and I would submit a recommendation that, if this be thought feasible, the incoming president appoint a prosecuting committee, to consist of one member from each district, whose duty it shall be to investigate, collect evidence, and prosecute any reported violations of the dental law in his district; and that the treasury of the society shall remunerate him, in part, at least, for the time spent in what is undoubtedly for the good of the entire dental profession of Massachusetts.

Another recommendation that I wish to make is that the time of the annual meeting be changed. Coming as it does during the first week of June, the Society is deprived of the advantage of hearing from a great number of the representative members of the profession, both from this State and others, who are connected with dental colleges and are unable to give any time to other than college matters at this the examination and commencement time. There are also many of our leading men who choose this season to go abroad, whereas, were the annual meeting held in the winter or spring, there would be less difficulty in securing material and a fuller attendance insured. Some of our members from the middle and western parts of the State have also expressed a desire to have the annual meeting held occasionally outside of Boston, to accommodate those living in the extreme western part of the State, who would be much more fully represented and whose district societies would be augmented in numbers if the annual meeting were held periodically in the central part of Massachusetts,—perhaps when the presiding officer was chosen from those districts,—and I would accordingly recommend that this idea be considered by the Society and acted upon if thought desirable.

In this progressive age of submarine boats, horseless carriages, wireless telegraphy, liquid air, and the great achievements of army and navy, do not, I beseech you, let our own chosen profession of dentistry be one of those to retrograde, as it of necessity must if not advanced. With fifteen hundred dentists in the State of Massachusetts, it is a reproach to the profession that there are less than two hundred and fifty members in the State Society. There should be at least a thousand. If they do not join because they do not consider themselves intellectually or ethically fit to associate with the members, then let them strive to become so. If it be merely from lethargy and indifference, let them realize that whatever advances their profession must necessarily raise their own individual importance in the community. If it is because they are so sordid and mercenary that they begrudge a few hours and a few dollars yearly, they should know that the more the profession is elevated the greater respect for it is accorded by the laity, and the value of services becomes more fully appreciated and will be remunerated accordingly, so that, after all, the pecuniary loss so much feared will turn to gain. There are many members, and some who are not members, who attend the annual meetings, taking all and giving nothing in return, carping critics who sneer at this or that meeting as a failure, throwing mud at those who at least have made an honest effort to do something and are therefore immeasurably superior to their detractors, who shirk all responsibility and ridicule all that is earnest, not even giving the encouragement of membership.

What becomes of all our younger graduates that are yearly let loose upon the community? Why do we not hear from them as joining the State Society? The first object a young man should have after graduating and passing the State Board of Examiners should be to become a member of his State Society and assure his standing in the profession, if not also in the community. As the Society grows in members it will also grow in importance and power, and membership will be recognized as a criterion of the standing of all practitioners in the State.

Every dentist who belongs to any other society and does not also belong to the State Society is not only helping to retard the advance of the profession, but is also standing in his own light, as the larger and more influential the latter becomes, the more important are the individuals composing it in the eyes of the laity. There are undoubtedly a great many reputable practitioners in Massachusetts who apparently have never heard of the Massachusetts Dental Society, or, if they have, only possess a very vague idea of it and its objects, some of them thinking it only a means of advertising the few who really take hold and labor for the welfare of the Society as well as the profession, trying to guard the latter from the downfall to a trade, which would speedily happen were it not for the dental literature and societies. If each member of this Society should appoint himself a committee to secure the membership of two or three reputable dentists, more would be accomplished towards the end in view than could be done in any other way. If members would also willingly serve on committees, it would be a great aid. At the last meeting of the South Metropolitan District Society it was impossible to secure from the very slim attendance a working force of officers and committees for the ensuing year. As all members expect to gain something by their membership, why should they refuse to accept their share of the burden?

With this final appeal to consider what has really become a very serious matter, and heartily thanking those who have so generously given their time and study to help make this meeting an interesting one, I will not trespass further upon your time and attention.

### DISCUSSION OF PRESIDENT'S ADDRESS.

*Dr. Geo. A. Maxfield* (Holyoke, Mass.).—I do not want to be identified as one of those who can see but one side of a question, but it is a great pity that the whole number of dentists in Massachusetts do not belong to the Massachusetts Dental Society, and they are wrong when they think the Society is more for the aggrandizement of the officers and of the few who take an active part. It is more for their benefit than for ours, and for the solution of some problems which we must all sooner or later solve.

While the reorganization of the Society in districts is not a failure, it is not the success that we hoped it would be; but it is very certain if we will work along on the lines we are already on it will prove a great success. There must be some plan outlined in some way whereby we can create renewed interest in our district societies. I do not think it any surprise to any of us that members do not take hold of these matters in better shape than they do, for I doubt if they appreciate the amount of benefit these societies confer upon them. Any of them who know the history of the Dental Protective Association, and know how much that has saved the pockets of the members of our profession, also know that there are but a little over three thousand members in the United States, at a nominal expense of ten dollars each. Now, if the members of the dental profession will not go into anything to help their pockets, it is hard to get them to go into anything that will not help them in dollars and cents. It is hard to get them into such societies as ours. We cannot make it a success unless there are more men who are willing to sacrifice something in order to make it a success.

The President has made some excellent recommendations, and I move that a committee of three be appointed by the President to take these recommendations under consideration and make a report at the councillors' meeting to be held to-morrow forenoon.

*Dr. A. J. Flanagan* (Springfield, Mass.).—That portion of the President's address referring to the holding of meetings in the central and western part of the State especially appeals to the Valley District, as also his remarks in relation to the enforcement of the dental law. We believe in the western part of the State that if we have a law, and it is a good law, we should all help to support it. We also believe that the Dental Board of Massachusetts can assist us in enforcing the law. Now that the laws are enacted, we should all help out their enforcement. I thoroughly believe that something should be done by the State Society to enforce the laws at this time, and I heartily agree with Dr. Maxfield that a committee be appointed, and, if necessary, that we should go down in our pockets and aid the committee in every way to secure the enforcement of said law.

*President H. S. Draper.*—I would like to hear from Dr. Boardman.

*Dr. Waldo E. Boardman* (Boston, Mass.).—I will say that I consulted with a member of the Board of Registration of Vermont, who informs me that each county has a prosecuting attorney, termed State's Attorney, and complaint is entered with him as in any criminal proceeding. The State Society has a man whom they term a State prosecutor, who serves without compensation, and if you do not wish to make the complaint yourself, you can write to him.

*President H. S. Draper.*—There would be more interest taken if the men were taken from our own Society, and I would like to inquire of Dr. Maxfield if he would like to have that committee appointed now.

*Dr. Geo. A. Maxfield.*—I think the quicker it is appointed the better.

*President H. S. Draper.*—The chair will appoint on that committee Drs. Waldo E. Boardman, D.D.S., George A. Maxfield, and L. D. Shepard.

**(To be continued.)**

